# BARRIERS AND FACILITATORS TO SOCIAL PARTICIPATION WHEN AGING WITH A LONG-TERM DISABILITY: A SCOPING STUDY

**DOI:** 10.1093/geroni/igae098.4126

**Published:** 2024-12-31

**Authors:** Samuel Turcotte, Sirine Kheroua, Angéline Labbé, Mia Lapointe, Manh Hung Nguyen, Megan Veilleux, Pascale Simard, Melanie Levasseur

**Affiliations:** Université Laval, Québec, Quebec, Canada; Université Laval, Québec, Quebec, Canada; Université Laval, Québec, Quebec, Canada; Université Laval, Québec, Quebec, Canada; Université Laval, Québec, Quebec, Canada; Université Laval, Québec, Quebec, Canada; Université Laval, Québec, Quebec, Canada; Université de Sherbrooke, Sherbrooke, Quebec, Canada

## Abstract

**Introduction:**

Supporting the social participation of individuals aging with a long-term neurological disability contributes to active and healthy aging. However, knowledge about factors influencing the social participation of this population remains scarce.

**Objectives:**

Integrate knowledge regarding the barriers and facilitators to social participation for people aging with a long-term neurological disability.

**Methods:**

A scoping review following the Joanna Briggs Institute guidelines was conducted across four databases (MEDLINE, CINAHL, PsycInfo, and EMBASE). Analysis relied on the Human Development Model - Disability Creation Process (HDM-DCP). This study is reported according to the PRISMA extension for scoping review (PRISMA-ScR).

**Results:**

Eighteen articles (8 quantitatives and 10 qualitatives) representing 1,319 participants and eight neurological conditions (e.g., stroke, multiple sclerosis, traumatic brain injury, spinal cord injury, aphasia, post-polio, spina bifida, and cerebral palsy) were included. Participants’ average age ranged from 53.4 to 72 years and the average time since their disability ranged from 5 to 46.26 years. A total of 26 barriers and 42 facilitators to social participation were identified. The most recurrent barriers were associated with the organic system (e.g., presence of comorbidities) and the societal environment (e.g., transportation accessibility). The most frequent facilitators included identity factors (e.g., acceptance of one’s condition) and the community environment (e.g., available social participation opportunities).

**Conclusion:**

This study lays the groundwork for developing interventions aimed at promoting active and fulfilling aging among older adults with long-term disabilities. Supporting social participation for this population should involve harnessing individual strengths and environmental resources as well as eliminating environmental systematic barriers.

